# Unfractionated heparin reverses aspirin inhibition of platelets during coronary artery bypass graft surgery

**DOI:** 10.1038/s41598-024-58005-x

**Published:** 2024-04-13

**Authors:** Robert E. Turnbull, Azhar Hafeez, Katrin N. Sander, David A. Barrett, Gavin J. Murphy, Alison H. Goodall

**Affiliations:** 1 Department of Cardiovascular Sciences, University of Leicester and NIHR Cardiovascular Biomedical Research Centre, Glenfield Hospital, Leicester, UK; 2 Centre for Analytical Bioscience, Advanced Materials and Healthcare Division, School of Pharmacy, University of Nottingham, Nottingham, UK; 3 Present Address: The Leicester Institute of Structural and Chemical Biology and Department of Molecular and Cell Biology, University of Leicester, Lancaster Road, Leicester, LE1 7HB UK; 4 Present Address: Division of Clinical Medicine, School of Medicine and Population Health, University of Sheffield, Sheffield, S10 2HQ UK

**Keywords:** Platelets, Cardiovascular diseases, Platelets, Drug delivery

## Abstract

Unfractionated heparin (UFH) is an effective antithrombotic during surgery but has known adverse effects, in particular on platelets. A marked increase in platelet responsiveness has previously been observed in patients within minutes of receiving UFH, despite adequate inhibition by aspirin prior to heparin. We studied this phenomenon in patients undergoing cardiac artery bypass grafting (n = 17) to determine whether the effects of heparin were systemic or platelet-specific. All patients’ platelets were fully inhibited by aspirin prior to surgery, but within 3 min of receiving heparin spontaneous aggregation and responses to arachidonic acid (AA) and ADP increased significantly (p ≥ 0.0002), and activated platelets were found in the circulation. While there was no rise in thromboxane in the plasma following heparin, levels of the major platelet 12-lipoxygenase product, 12-HETE, rose significantly. Mixing experiments demonstrated that the changes caused by heparin resided primarily in the platelets, while addition of AA pathway inhibitors, and analysis of oxylipins provided evidence that, following heparin, aggregating platelets regained their ability to synthesise thromboxane. These findings highlight potentially unrecognised pro-thrombotic and pro-inflammatory changes during CABG surgery, and provide further evidence of adverse effects associated with UFH.

## Introduction

Patients undergoing surgery routinely receive aspirin and unfractionated heparin to prevent thrombotic complications by inhibiting, respectively, platelet activation and thrombin’s proteolytic activity.

Activation of platelets releases arachidonic acid (AA) from platelet membrane phospholipids^1^. Aspirin binds irreversibly to cyclooxygenase-1 (COX-1) preventing the metabolism of AA to the secondary platelet agonist thromboxane A2 (TXA2). Unbound TXA2 is unstable and rapidly converts to TXB2, which is the form that is normally measured. Lacking a nucleus, platelets are unable to synthesise COX-1 de novo and so aspirin inhibition lasts for the lifetime of the platelet (8–10 days).

However, aspirin is not always effective in all patients, resulting in the phenomenon of aspirin resistance. Estimates of this phenomenon based on clinical endpoints in CVD patients vary, but it is now generally acknowledged that true aspirin resistance, in patients with confirmed drug compliance, should be based on laboratory assessment of platelet responses, or thromboxane generation in vivo or in vitro^2,3^. Using this definition, aspirin resistance is reported in ~ 25% of CVD patients, and is due to factors that include polymorphisms in COX-1 and other relevant proteins, up-regulation of non-platelet sources of thromboxane, and increased platelet turnover^4^, and is associated with higher cardiovascular risk^5^.

A much higher percentage (~ 70%) of patients undergoing Coronary Artery Bypass Grafting (CABG) are reported to experience a reduced response to aspirin in the days following surgery^6–9^. These findings, based on laboratory assessment, are seen one or more days post-surgery, and have been linked to increases in platelet count, suggesting that this decline in aspirin response is likely due to de novo synthesis of platelets.

Conversely, we have previously seen a transient, and reversible form of aspirin resistance in patients undergoing carotid endarterectomy (CEA) at a much earlier time point, which coincides with the administration of unfractionated heparin (UFH). Three minutes after heparin administration, platelets from patients who, prior to surgery had adequate aspirin inhibition of their platelets, showed significant increased aggregation in response to AA, ADP and the thrombin receptor agonist receptor peptide (TRAP), which returned to pre-heparin aspirin levels within 24 h without further aspirin administration^10,11^. The increased response to AA^10^ was unaffected by heparin type^12^. However, the response to ADP was significantly lower if patients received low molecular weight heparin (LMWH) compared to UFH^12^, suggesting different mechanisms underlying the increased responses to AA and ADP.

Thromboxane is not the only product of AA generated by activated platelets. They contain 12-lipoxygenase (12-LOX), which also utilises AA to generate a range of oxylipins^13,14^. The major product of this pathway is 12-HETE, which is released from platelets^15,16^ where it has paracrine effects on other vascular cells^17^. Platelets also generate low levels of oxylipins from AA via both COX-1 and 12-LOX, including 8-, 9-, 11-, 15- and 16-HETE^15,16^.

In addition to the products of AA and LA, the potent platelet agonist, Platelet Activating Factor (PAF), can be generated from lysophosphatidylcholine (LPC) liberated from membrane phosphatidylcholine^18^, which is rapidly acetylated to produce PAF^19^.

In previous studies of CEA patients we showed that after administration of unfractionated heparin, whilst the level of TXA2 in the patients’ plasma remained unchanged compared to their pre-heparin levels, 12-HETE in the plasma was significantly elevated post-heparin^20^, and we suggested a possible role for 12-HETE or other oxylipins or PAF in this heparin-induced transient aspirin resistance.

Here we have investigated this phenomenon further in patients undergoing coronary artery bypass graft (CABG) surgery and found it was not due to systemic factors released by heparin into the circulation, but rather to changes in the platelets themselves, and asked whether generation of oxylipins and other lipid mediators within the platelets might account for this phenomenon of transient aspirin resistance.

## Results

### Patients

**Figure 1:**
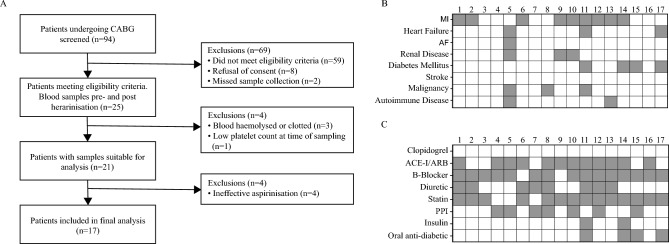
Patient recruitment and clinical data. (**A**) Study design and recruitment. (**B**) Past medical history and (**C**) current medication for the patients included in the study. Numbers indicate patient study ID. White boxes indicate no; grey boxes yes.

Of the 94 patients scheduled for CABG surgery during the study period 69 were excluded for not meeting eligibility criteria. Of these, 8 did not provide consent and 59 were either not taking aspirin before surgery, or were still on dual anti-platelet therapy, had a self-reported history of bleeding or easy bruising, clinical evidence of prior heparin induced thrombocytopenia, or had a platelet count outside the normal range. Sample collection was missed for a further 2 patients (Fig. 1a). Of the remaining 25, a further 4 were excluded after sample collection due to haemolysis or clotting in the blood samples (n = 3) or for failure to meet an inclusion criterion (low platelet count; n = 1). A further 4 patients were subsequently excluded from the analysis due to a sub-optimal response to aspirin prior to surgery (> 25% aggregation in response to AA). This number (4/25) is in line with the reported rate of ~ 25% laboratory-defined aspirin resistance in CVD patients^4^, but all responded to aspirin in vitro, suggesting non-compliance rather than true aspirin resistance (Supplementary Fig. 1).

The demographics of the 17 patients included in the study are shown in Table 1. They were aged between 56 and 83 years (mean of 68.5 years), all were male, and 16/17 were white northern Europeans; the remaining patient being of South Asian origin. Their BMIs ranged from 22.7 to 39 (mean of 31), with 7 classed as obese (≥ 30) and 5 as overweight (≥ 25). As illustrated in Fig. 1b, of the 17 patients who were included in the study, 13 had other co-morbidities including 9 who had a cardiac event prior to their presenting MI. None had a prior history of stroke. All were on 75 mg pd aspirin but patients taking a P2Y12 antagonist ceased this medication at least 7 days prior to surgery. None were taking any other anti-platelet drugs, but all were taking between 2 and 6 other medications (Fig. 1c).

**Table 1:** Demographics of patients completing the study.

Variable	Mean value (range) or n
Age in years	68.5 (56–83)
Male gender	17 (100%)
Ethnicity (white British: South Asian)	16:1 (94%)
BMI	29.4 (22.7–39.0)
Smoking status (current:Ex:None:unknown)	0:11:3:3

### Heparin increases platelet activation in patients undergoing CABG

**Figure 2:**
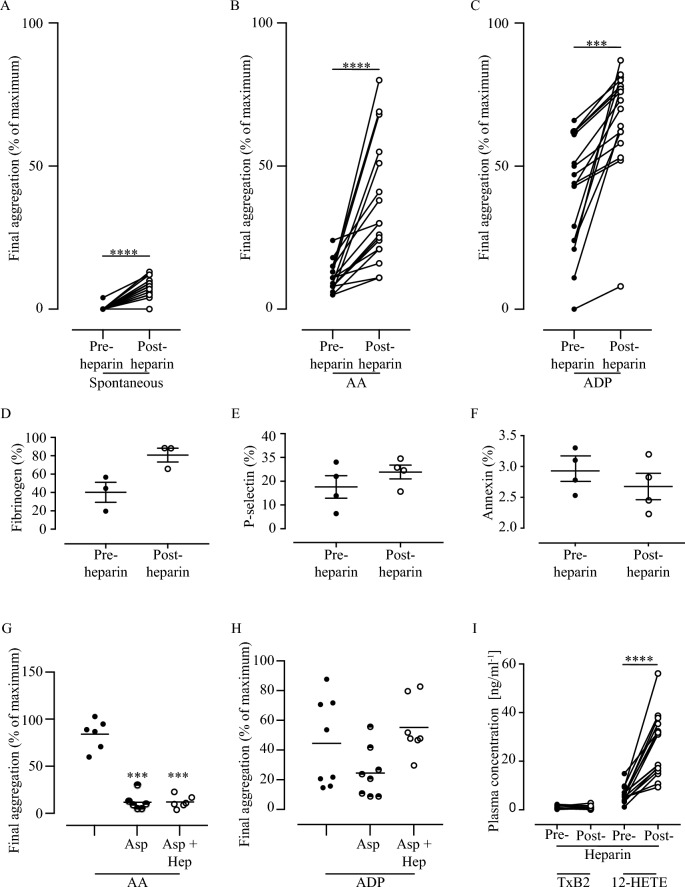
Heparin administration increases platelet reactivity and activation. (**A**–**C**) Aggregation of patients’ platelets pre- and post-heparin administration. (**A**) Spontaneous aggregation; (**B**) aggregation in response to 2 × 10^−3^ mol/l AA or (**C**) to 3.2 × 10^−6^ mol/l ADP. (**D**–**F**) Platelet activation detected by flow cytometry; (**D**) binding of fibrinogen to activated GPIIb-IIIa; (**E**) Expression of P-selectin as a marker of platelet degranulation; (**F**) binding of Annexin V as a marker of a procoagulant platelet surface. (**G**–**H**) Effect of addition of aspirin and heparin on aggregation of aspirinated platelets from healthy controls in response to (**G**) AA or (**H**) ADP. (**I**) Levels of TXB2 and 12-HETE measured by ELISA in patients’ plasma pre-and post-heparin. P-values calculated using Student’s paired T-test (***< 0.001, ****< 0.0001).

Prior to heparin infusion, none of the patients’ platelets showed any spontaneous platelet aggregation, all showed low (< 25%) aggregation in response to AA, and all showed only primary aggregation responses to ADP (Fig. 2a–c), indicating effective aspirinisation. However, in samples taken 3 min after infusion of unfractionated heparin platelet responsiveness increased significantly. There was a small (approximately twofold) and significant increase in spontaneous aggregation (p < 0.0001) (Fig. 2a), while aggregation to AA increased markedly in all patients (p < 0.0001) (Fig. 2b), as did the response to ADP (p = 0.0002) (Fig. 2c), with all patients showing both primary and secondary ADP responses.

As is evident from the data shown in Fig. 2 there was considerable variation in the patients’ platelet responses following heparin infusion, but only modest correlation between the subjects’ responses to each agonist pre-and post-heparin, or between agonists (r^2^ < 0.432; p > 0.01 for all. Supplementary Table 1). There were similar, modest associations between the patients’ BMI and their aggregatory responses (Supplementary Table 1), but as this relationship was seen both pre- and post-heparin, factors relating to BMI are unlikely to be causative in the post-heparin response.

Flow cytometric analysis of the platelets in the blood of a sub-set of patients showed an increased expression of markers of activation in vivo following heparin; a doubling of the percentage of platelets carrying fibrinogen (a marker of activation of the GPIIb-IIIa complex) and a small increase in P-selectin expression (a marker of platelet degranulation) (Fig. 2d,e). However, there was no increase in Annexin V binding; a marker of platelet procoagulant activity^21^ (Fig. 2f). While none of these changes reached statistical significance (p > 0.05 for all) this reflects the relatively small number of subjects analysed.

As is well established, addition of heparin in vitro, at a concentration of 2 units ml^−1^ (equivalent to the 10,000 unit bolus given to the patients) did not restore the response to AA in platelets from healthy subjects (n = 8) that had been pre-treated with 300 μM aspirin (Fig. 2g) to fully inhibit the platelet response. However, heparin was able to overcome the inhibition by aspirin when the aspirinated platelets from the healthy subjects were stimulated with ADP (Fig. 2h). This both confirms the known effects of heparin in vitro and demonstrates that this differs from the effects of heparin in vivo that is seen ex vivo in the patients’ platelets.

### Is the effect of heparin mediated through lipid intermediaries generated in vivo?

The increases in the platelet response and the increased basal activation status seen in the patients’ platelets following heparin were not accompanied by an increase in the level of TXB2 in the patients’ plasma (Fig. 2i), indicating that the effects of aspirin on COX-1 were not being overcome. However, the level of 12-HETE in the plasma increased significantly (Fig. 2i; p < 0.0001), as has been observed previously in other surgical patient groups^20^. These observations suggest mobilisation of AA in vivo, either from platelets or other cells, against a background of effective inhibition of platelet COX-1.

**Figure 3:**
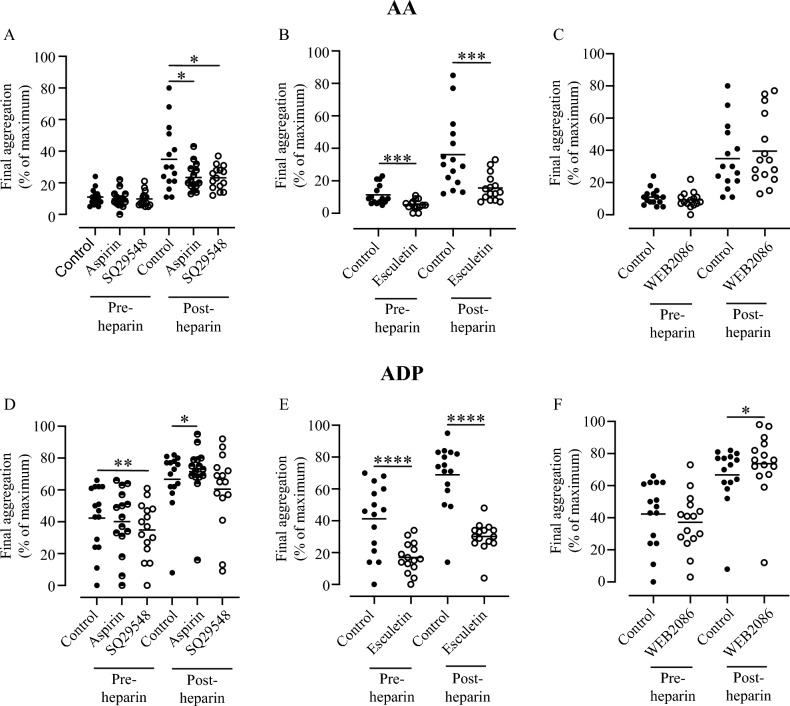
Role of AA-derived oxylipins in the post-heparin platelet response. The effect of 3 × 10^−4^ mol/l aspirin or 1 × 10^−6^ mol/l of the TP antagonist SQ29548 on the patients’ platelet aggregation in response pre and post-heparin administration to (**A**) AA or (**D**) ADP. The effect of 2 × 10^−5^ mol/l esculetin on the patients’ platelet aggregation in response to (**B**) AA or (**E**) ADP. The effect of 3 × 10^−5^ mol/l of the PAF receptor antagonist, WEB2086, on the patients’ platelet aggregation in response to (**C**) AA or (**F**) ADP aggregation. P-values calculated using the Student’s paired T-test (*< 0.05, **< 0.01, ***< 0.001, ****< 0.0001).

To explore this further, PRP from the patients’ pre- and post-heparin blood samples were incubated ex vivo with either additional aspirin or the thromboxane receptor antagonist SQ29548. While neither inhibitor had any effect on the aggregation response to AA prior to heparinisation, both caused a modest, but significant (~ 35%) reduction of the aggregation to AA seen post-heparin (Fig. 3a; control vs aspirin p = 0.025; control vs SQ29548 p = 0.0203). In contrast, neither of these inhibitors reduced the platelet responses to ADP (Fig. 3d), and indeed a slight increase was seen in the post-heparin response in the presence of further aspirin (p = 0.0207). This may explain why aspirin in vitro caused only partial inhibition of the post-heparin response to AA, which could be due to ADP released from platelets in vitro during the aggregation assay*.*

The 12-LOX inhibitor esculetin produced significant inhibition of the response to both AA, (Fig. 3b), and ADP (Fig. 3e) (p ≤ 0.001 for all) in both the pre- and post-heparin samples, and parallels the effects of this concentration of esculetin in vitro on non-aspirated platelets, where it completely blocks platelet aggregation without affecting degranulation as well as blocking the production of 12-HETE^16^. However, since esculetin is a non-specific inhibitor, acting primarily though anti-oxidant mechanisms that blocks the generation of virtually all AA metabolites by platelets, including TXA2^16^ these data do not prove a role for 12-HETE. While a more specific 12-LOX inhibitor (ML355) has been developed, we have recently demonstrated that this also blocks the generation of TXA2 and other COX products by platelets^16^ and so it would not have proved useful in the present study to tease out the role of 12-HETE.

The alternative lipid platelet agonist PAF also could not be identified as the cause of the increased platelet response post-heparin since the PAF antagonist WEB2086 had no effect on the response to AA (Fig. 3c) (p = 0.1156) and, like aspirin, caused a small but significant increase in response to ADP (Fig. 3f) (p = 0.0384).

None of the changes in platelet reactivity is likely to be due to the release of newly formed platelets from megakaryocytes, nor from mobilisation of platelets from the spleen, since platelet counts did not increase, but rather showed a small decrease (− 6.5%; p = 0.012) following heparin. There was also a small reduction in haemoglobin (− 8%; p = 0.039), consistent with modest blood loss and/or haemodilution due to fluid replacement, and a small, non-significant increase in leucocyte count (+ 4.6%; p = 0.069), consistent with an inflammatory response to surgery (Supplementary Fig. 2).

### The effect of heparin resides in the platelets rather than the plasma

Since heparin is known to release many molecules from both cells and lipoproteins^22^ we next explored whether the changes seen in the platelets were due to other factors that may be released into the plasma following heparin infusion, or whether the changes reside in the platelets themselves.

**Figure 4:**
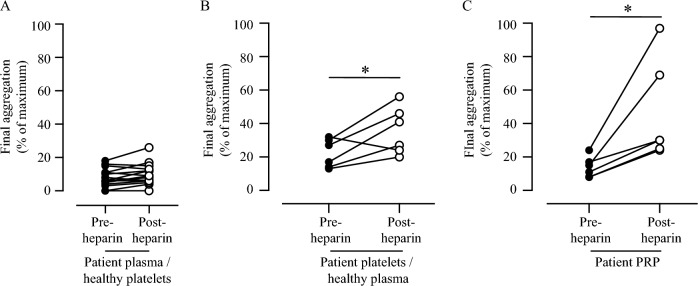
Reversal of aspirin by heparin resides in the patients’ platelets. Aggregation in response to AA. (**A**) Washed platelets from healthy volunteers, pre-incubated with aspirin and resuspended in plasma collected from patient’s pre- and post-heparin (n = 15). (**B**) Aggregation of washed patients’ platelets in plasma from healthy volunteers (n = 6). (**C**) Aggregation of the same patients’ platelets PRP (n = 6). P-values calculated using the Student’s paired T-test (*< 0.05).

When washed, aspirinated platelets from healthy subjects were mixed with plasma from the patients there was no significant aggregation in response to AA with either the pre- or post-heparin plasma samples (Fig. 4a) demonstrating that the patients’ plasma did not contain material that could account for the increased platelet response seen following heparin treatment. Conversely, when washed patients’ platelets were incubated with plasma from healthy subjects there was a significant increase in the aggregation response to AA by the platelets taken after heparin, compared to the patients’ pre-heparin platelet samples (Fig. 4b). Although this did not quite reach the level of increase seen when patients PRP was treated with AA (i.e. in the presence of autologous plasma; Fig. 4c), it does indicate that the pro-aggregatory effect is due to changes in the patients’ platelets that can overcome the effects of aspirin, rather than factors present in the patients’ plasma.

### Heparin-induced changes to oxylipin generation in platelets

**Figure 5:**
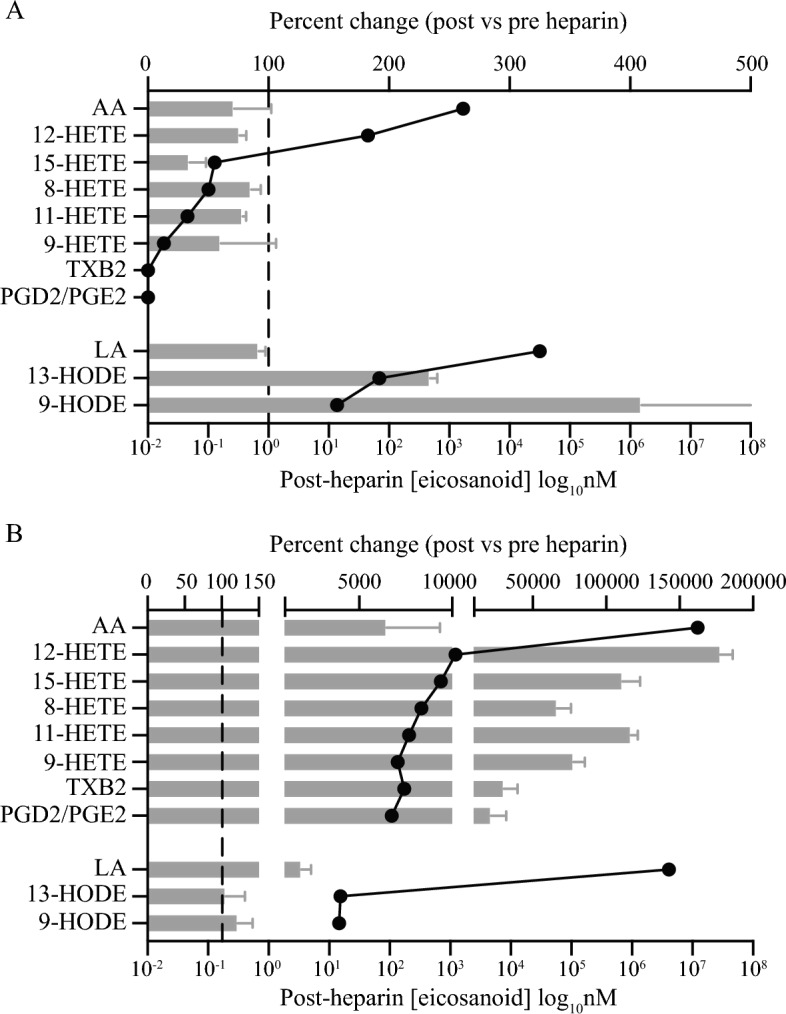
Changes in oxylipin profiles of the patients’ platelets following heparin infusion. (**A**) Grey bars show the percentage change in the levels of oxylipins in the patients’ platelets following heparin administration, measured by LC–MS/MS. Upper x-axis; mean ± SEM. Filled symbols show actual levels of oxylipins in the patients’ platelets post-heparin (nM) (lower x-axis). n = 2. (**B**) Grey bars show the percentage change in the levels of oxylipins released in vitro from spontaneously aggregating platelets following heparin administration, measured by LC–MS/MS (upper x-axis; mean ± SEM) (n = 2). Filled symbols show actual levels of oxylipins released from the spontaneously aggregating platelets post-heparin (nM) (lower x-axis). Platelets from 8 patients were randomly allocated to 2 pools prior to analysis and each pool was analysed in duplicate.

While generation of TXB2 from AA via COX-1 was effectively blocked in the patients’ platelets in vivo, other oxylipins can be generated via AA redirected through 12-LOX, or via autoxidation of AA^15,16^, some of which have been reported to activate platelets. This is illustrated by the significant increase in 12-HETE seen in the patients’ plasma post-heparin. However, since the patients’ post-heparin plasma did not induce activation when mixed with healthy donor platelets it is unlikely that this, or any other AA metabolites in the patients’ plasma are the causative agents. To test whether heparin could affect changes in the oxylipin profile within the platelets themselves, oxylipins were extracted from platelets before and after heparin infusion and analysed by mass spectrometry (Fig. 5a). Interestingly, levels of all HETE isoforms were either unchanged, or slightly reduced in the platelets following heparin infusion, while TXB2 remained undetectable in the platelets both pre- and post-heparin, as did the prostaglandins PGD and PGE that are also generated via COX-1. However, there were large (400–500%) increases in the levels of the LA products 9- and 13-HODE (Fig. 5b) confirming that PLA2-mediated fatty acid release from the platelet membrane phospholipids was occurring. These LA-derived HODEs are unlikely to account for the changes seen in the patients’ platelets since there is no evidence that HODEs can induce platelet aggregation; rather they have been reported to inhibit platelet activation^23,24^.

Surprisingly, while there was no increase in oxylipins within the platelets, the platelets taken from the patients after heparin infusion and allowed to spontaneously aggregate ex vivo were capable of generating and releasing significant amounts of all the AA-derived oxylipins as well as AA (Fig. 5b). This included the appearance of TXB2 as well as PGE2 and PGD2 that are also generated through COX-1. Although levels of LA also increased neither LA itself, or the LA derived HODEs were released from the platelets.

## Discussion

Patients recruited sequentially from those undergoing scheduled CABG, who met the inclusion/exclusion criteria, and who were demonstrated, by measurement of their platelet response to AA and ADP to be adequately aspirinated, all showed a rapid (within 3 min) rise in their platelet responses to AA and ADP. This is in line with our previous findings in patients undergoing CEA^11^ and confirms that the effect is linked to the infusion of unfractionated heparin. This rapid effect, which returns to normal within 24 h without further aspirin treatment^11^, is distinct from the reported increase in platelet aspirin resistance seen in up to 70% of patients in the days following CABG, and attributed to de novo production of new (and therefore aspirin-naive) platelets^6–9^.

This also differs from platelet activation seen in those patients who develop heparin-induced thrombocytopenia (HIT), arguably the most serious adverse reaction to heparin, which is due to an auto-immune response that appears in patients several days after receiving UFH^25^. This activation is caused by complexes of heparin and Platelet Factor 4 (PF4) that bind to and activate the platelet through their Fcγ receptors. It results in more extensive platelet activation than that seen in our post-heparin samples and leads to their destruction and release of procoagulant platelet-derived MVs, with the resultant seemingly anomalous combination of thrombocytopenia and thrombosis that characterises HIT. Both the rapid effect seen in the present study, and the fact that Annexin V, the marker of procoagulant platelets, was not increased 3 min after the patients received heparin, indicates that HIT mechanisms are not involved in the changes reported here. None of the patients in this current study had a history of HIT, and there were no reports of HIT developing after surgery, although this was not formally assessed.

One of the questions we sought to address in the current study was whether the effects seen were due to factors released into the plasma by heparin, or to changes within the platelets themselves. We have previously shown that the heparin-induced increase in the platelet response to AA is not due to the presence of platelet agonists ADP or thrombin in the plasma^10^. Here our data also demonstrates that the potent platelet agonist PAF is also not responsible for the effects seen since neither the response to AA nor to ADP was inhibited by the PAF antagonist WEB2086. We also clearly demonstrate that the effect of heparin is not caused directly by other factors that become elevated in the patients’ plasma, including 12-HETE as previously hypothesised^20^, but resides in the platelets themselves, since post-heparin plasma from the patients did not replicate the effects seen in the patients when added to aspirinated platelets from healthy volunteers.

However, the observation that the increased response to AA in the patients’ platelets following heparin infusion was inhibited by ~ 35% by adding further aspirin, or the TP antagonist SQ29548 in vitro, suggests that the platelets re/gain the ability to generate TXA2 or a related platelet-stimulatory oxylipin. This was substantiated by the increased levels of the COX-metabolite TXB2 and prostanoids released by the platelets after heparin infusion, following spontaneous aggregation. Heparin is known to release lipoprotein and hepatic lipases into the circulation^26–28^ and we have shown this to be the case in surgical patients receiving both UFH and LMWH^20^. These lipases can release fatty acids, including AA and LA, from a range of sources, including platelets, therefore following heparin infusion, higher levels of AA and LA may be available to support increased synthesis of eicosanoids and other oxylipins within the platelets upon stimulation. This may also explain why in vitro aspirin caused only partial inhibition of the post-heparin response to AA, which could be due to either the generation of other lipoxygenases in the platelets (e.g. 15-HETE) that are not completely inhibited by aspirin^15,16^, and/or to ADP released in vitro during the assay, since the post-heparin ADP response was unaffected by adding aspirin (Fig. 3D).

Taken together our findings point to a direct change in the platelets induced by heparin. Heparin is known to be able to augment the effect of other stimuli via cross-linking the fibrinogen receptor through PKCγ-mediated outside-in signalling, but requires another stimulus to induce aggregation^29,30^. Therefore, infusion of heparin could lead to the activation of some platelets in vivo*,* as well as their sensitisation to the subsequent effect of other agonists such as ADP in vivo and in vitro, both of which were demonstrated in the present study. The lack of effect of heparin in vitro on platelets from healthy subjects’ points to other confounding factors in the patients that support a degree of activation in vivo, which from our data could be due to increased availability of AA.

Our data also highlights the significant degree of inter-patient variability in the platelet response, which points to the potential benefit for more personalised antiplatelet therapy. It is of note that the patients in this study received a weight-independent dose of 10,000 IU heparin, which may explain the modest association of their BMI with their post-heparin platelet responses.

In summary, patients undergoing CABG surgery show an increased aggregatory reponse, both spontaneous and in response to AA and ADP, following heparin infusion that could increase the risk of thrombotic and inflammatory response in these patients. The changes induced by heparin that render the platelets more responsive lie in the platelets themselves. The mechanism of the increased response to AA and ADP occur via different mechanism since the latter is not inhibited by aspirin or a TP antagonist. Finally, although heparin infusion leads to significantly increased plasma levels of the platelet 12-LOX products our evidence suggests that this is unlikely to be responsible for the increases in platelet activation. 12 HETE is, however, known to have a range of pro-inflammatory roles both in vitro and in vivo that may, at least in part, account for some of the inflammatory changes seen in surgical patients^31^.

Importantly our findings raise the question of whether current antithrombotic therapy in surgery is optimal, and whether, with the advent of newer, direct antithrombotics, the use of UFH should be reconsidered. Similarly, since some of the inhibition of aspirin can be overcome by heparin, consideration of dual antiplatelet therapy with a P2Y12 antagonist could be of benefit. P2Y12 antagonists that block the ADP response are now widely used in other surgical conditions, for example in patients undergoing carotid endarterectomy surgery following clinical trials that demonstrated a reduced intra-surgical embolization without increased bleeding^12,32^. Whether this would be an effective strategy in patients undergoing more invasive CABG surgery warrants carefully controlled clinical investigation.

## Methods

### Reagents

Oxylipin standards for LC–MS/MS were purchased from Cayman Chemical (Cayman Islands) or from Enzo Life Sciences (Exeter, UK). The thromboxane receptor antagonist (SQ29548) was from Alexis Biochemicals Corporation (San Diego, USA). FITC-conjugated rabbit anti-human fibrinogen was from Dako, (UK, Ltd), FITC-conjugated anti-CD62P monoclonal antibody and an isotype control were from R&D Systems (Abingdon, UK) as was Annexin V FITC. Unless otherwise stated, all other reagents were purchased from Sigma Aldrich (Dorset, UK).

### Study design

The study of patients undergoing CABG was given ethical approval by NRES Committee East Midlands, Derby (REC reference 13/EM/0355) and performed in accordance with guidelines outlined in the Declaration of Helsinki. Male patients over the age of 18 who had been admitted to the Glenfield Hospital for elective CABG surgery were screened, and those eligible provided written informed consent prior to surgery. All patients received 75 mg aspirin daily for a minimum of 14 pre-operative days but were asked to stop taking P2Y12 antagonists, if prescribed, at least 7 days prior to surgery. Surgery was carried out according to standard clinical practice. During surgery, following opening of the chest cavity and harvesting of vessels, and prior to connection to cardiac bypass, the anaesthetised patients received an intravenous, patient-independent dose of 10,000U unfractionated heparin (UFH).

Exclusion criteria were; inability to provide consent, past history of a bleeding condition or heparin-induced thrombocytopenia (HIT), an abnormal platelet count (< 150 × 10^9^/l or > 500 × 10^9^/l), dual anti-platelet therapy within 7 days of surgery, inability to receive aspirin or heparin, or a poor response to aspirin (aggregation > 25% in response to AA).

### Blood sampling and processing

Blood was collected from the patients at two time-points, based on our previous observation of the effect of heparin: immediately before the administration of heparin then 3 min following intravenous infusion of UFH. Samples were taken from an arterial line into a syringe, immediately transferred to 0.105 M tri-sodium citrate anti-coagulant tubes (Becton Dickinson, Oxford, UK) and processed within 10 min.

Blood from healthy volunteers, used to establish dose ranges of agonists and inhibitors, and for specific in vitro experiments, was collected from the antecubital fossa by venepuncture using a 21-gauge butterfly needle into 0.105 M tri-sodium citrate vacutainers (Becton Dickinson, Oxford, UK). Volunteers had been free of medication for at least 14 days prior to donation and provided written informed consent. Ethical approval for these samples was granted by the University of Leicester’s Committee for Research Ethics Concerning Human Subjects (ref: ahg5-97b2).

To prepare platelet rich plasma (PRP) blood samples were centrifuged at 160*g* for 20 min.

To prepare washed platelets, acid citrate dextrose (0.085 M tri-sodium citrate, 0.071 M citric acid, 0.11 M glucose, pH 6.3) was added to citrated blood before centrifugation at 160 g for 20 min. The resulting PRP was transferred to a fresh tube and platelets pelleted by centrifugation at 600*g* for 15 min followed by two washes in HBS pH 6.0 (10 mM HEPES; 150 mM NaCl; 5 mM LiCl; 1 mM MgSO_4_) and re-suspension in 1 ml HBS pH 7.4. Freshly prepared prostacyclin was added at a final concentration of 0.57 μM before each centrifugation to minimise platelet activation. Following the final centrifugation step the washed platelets were either pelleted and stored at − 80 °C prior to oxylipin extraction, or re-suspended in either HBS pH 7.4 or platelet poor plasma at concentrations between 200 and 400 × 10^6^ ml^−1^ for aggregation studies. Prior to aggregometry the final platelet suspensions were left at room temperature for 30 min to allow the prostacyclin to decay.

Platelet free plasma (PPP) was obtained by centrifuging citrated blood at 1000 g for 30 min. Autologous plasma was used to set the baseline for the aggregometry studies, and the remainder was stored in aliquots at − 80 °C for further analysis.

### Platelet aggregometry

Platelet aggregation; spontaneous, and in response to AA (final concentration 2 × 10^−3^ mol/l) and ADP (final concentration 3.2 × 10^−6^ mol/l), was measured by Born-type aggregometry using a PAP8 aggregometer (BioData Corporation Horsham, PA, USA). Inhibitors were added to the PRP at final concentrations of; aspirin (3 × 10^−4^ mol/l); thromboxane receptor antagonist (SQ29548) (1 × 10^−6^ mol/l); platelet activating factor antagonist (WEB2086) (3 × 10^−5^ mol/l) or esculetin (2 × 10^−5^ mol/l)) and incubated at 37 °C for 10 min with stirring prior to initiating platelet aggregation. Agonist concentrations were selected to give maximum aggregation and inhibitors were used at levels that provided maximum inhibition of aggregation for each agonist, tested in 6 separate healthy donors (data not shown).

### Platelet flow cytometry

Patients’ platelets were analysed for markers of activation using a whole blood flow cytometric method essentially as described previously^10,32,33^. Briefly, 5 µl of citrated blood were added to 50 µl of HEPES-buffered saline (HBS; 10 mM HEPES; 150 mM NaCl; 5 mM LiCl; 1 mM MgSO_4_; pH 6.0) containing either 5 µl of a FITC-conjugated rabbit anti-human fibrinogen (Dako) or a FITC-conjugated anti-human CD62P monoclonal antibody (R&D Systems) that detects surface expression of P-selectin; a marker of platelet degranulation. After 20 min incubation at 20–22 °C samples were diluted 100-fold with 0.2% (v/v) formyl saline and analysed within 2 h in a Gallios flow cytometer (Beckman Coulter, Milton Keynes, UK). Negative controls were set at 2% + ve (Supplementary Fig. 3). For fibrinogen binding the negative control comprised a parallel sample prepared in the presence of 2 mM EDTA to chelate calcium and thereby disrupt the binding of fibrinogen to the GPIIb-IIIa integrin. For P-selectin the negative control was an isotype control mouse antibody. Annexin V binding used a similar assay but with 2 mM Ca^2+^ and 0.5 mg ml^−1^ GPRP peptide added to the HBS to allow, respectively, Annexin V binding to procoagulant phospholipids and to prevent clotting of the plasma. The negative control for these samples comprised a parallel sample that lacked calcium (Supplementary Fig. 3).

### Quantification of platelet oxylipins

The levels of TXB2 and 12-HETE in the patients’ plasma were measured by ELISA (Enzo Life Sciences) according to the manufacturer’s instructions.

A more comprehensive analysis of the eicosanoids and other oxylipins was conducted by LC–MS/MS, essentially as described previously^16,34^. Lipids were extracted from washed platelets from matched pre- and post-heparin from two of the patients and from plasma isolated from the aggregometry cuvettes following spontaneous aggregation of the post-heparin PRP samples from 8 of the patients. Due to the small volumes available these plasma samples were randomly pooled into two batches and extracted in duplicate.

Internal controls (AA-d8, PGD2-d4 and 15(S)-HETE-d8) and the antioxidant, butylated hydroxytoluene (BHT), were added to each sample before extraction in 1 M acetic acid: propan-2-ol: hexane 1:10:15 (v:v:v) followed by hexane. Following evaporation of the hexane under a stream of nitrogen the oxylipins were reconstituted in 70% (v/v) ethanol for LC–MS/MS analysis. Quality controls, blanks and standards were extracted alongside the samples and analysed in parallel.

Samples were analysed using a 4000 QTRAP® system (AB SCIEX, Framingham, USA) with a Turbo V™ Ion source with a TurbolonSpray probe (AB SCIEX) coupled to a high-performance liquid chromatography system (Shimadzu, Milton Keynes, UK) as previously described^16^. Fragmented ion spectra were collected, and the data was analysed using the Analyst software (AB SCIEX).

### Statistical analysis

Data shown represent either the mean or mean ± SEM. For analysis between the pre- and post-heparin samples p-values were calculated using Student’s paired t-test whilst multiple comparisons were calculated using a 2-way ANOVA with Sidak’s correction for multiple comparisons.

### Supplementary Information

Supplementary Information.

## Data Availability

The datasets used and analysed during the current study are available from the corresponding author on reasonable request.

## References

[1] Dennis EA, Norris PC (2015). Eicosanoid storm in infection and inflammation. Nat. Rev. Immunol..

[2] Floyd CN, Ferro A (2014). Mechanisms of aspirin resistance. Pharmacol. Ther..

[3] Hankey GJ, Eikelboom JW (2006). Aspirin resistance. Lancet.

[4] Ebrahimi P (2020). Prevalence rate of laboratory defined aspirin resistance in cardiovascular disease patients: A systematic review and meta-analysis. Caspian J. Intern. Med..

[5] Wisman PP (2014). Platelet-reactivity tests identify patients at risk of secondary cardiovascular events: A systematic review and meta-analysis. J. Thromb. Haemost..

[6] Bednar F (2009). Aspirin is insufficient in inhibition of platelet aggregation and thromboxane formation early after coronary artery bypass surgery. J. Thromb. Thrombolysis.

[7] Hovens MM (2007). Prevalence of persistent platelet reactivity despite use of aspirin: A systematic review. Am. Heart J..

[8] Wand S (2018). The prevalence and clinical relevance of ASA nonresponse after cardiac surgery: A prospective bicentric study. Clin. Appl. Thromb. Hemost..

[9] Zimmermann N (2007). Aspirin-induced platelet inhibition in patients undergoing cardiac surgery. Platelets.

[10] Payne DA (2004). Platelet inhibition by aspirin is diminished in patients during carotid surgery: A form of transient aspirin resistance?. Thromb. Haemost..

[11] Webster SE (2004). Anti-platelet effect of aspirin is substantially reduced after administration of heparin during carotid endarterectomy. J. Vasc. Surg..

[12] McMahon GS (2009). Low molecular weight heparin significantly reduces embolisation after carotid endarterectomy—A randomised controlled trial. Eur. J. Vasc. Endovasc. Surg..

[13] Hamberg M, Samuelsson B (1974). Prostaglandin endoperoxides. Novel transformations of arachidonic acid in human platelets. Proc. Natl. Acad. Sci. USA.

[14] Mitchell JA, Warner TD (2006). COX isoforms in the cardiovascular system: understanding the activities of non-steroidal anti-inflammatory drugs. Nat. Rev. Drug Discov..

[15] Rauzi F (2016). Aspirin inhibits the production of proangiogenic 15(S)-HETE by platelet cyclooxygenase-1. FASEB J..

[16] Turnbull RE, Sander KN, Turnbull J, Barrett DA, Goodall AH (2022). Profiling oxylipins released from human platelets activated through the GPVI collagen receptor. Prostaglandins Other Lipid Mediat..

[17] Kulkarni A, Nadler JL, Mirmira RG, Casimiro I (2021). Regulation of tissue inflammation by 12-lipoxygenases. Biomolecules..

[18] Kita Y, Shindou H, Shimizu T (1864). Cytosolic phospholipase A(2) and lysophospholipid acyltransferases. Biochim. Biophys. Acta Mol. Cell Biol. Lipids.

[19] Chesney CM, Pifer DD, Byers LW, Muirhead EE (1982). Effect of platelet-activating factor (PAF) on human platelets. Blood.

[20] McMahon GS, Jones CI, Hayes PD, Naylor AR, Goodall AH (2013). Transient heparin-induced platelet activation linked to generation of platelet 12-lipoxygenase. Findings from a randomised controlled trial. Thromb. Haemost..

[21] Dachary-Prigent J, Freyssinet JM, Pasquet JM, Carron JC, Nurden AT (1993). Annexin V as a probe of aminophospholipid exposure and platelet membrane vesiculation: A flow cytometry study showing a role for free sulfhydryl groups. Blood.

[22] Myrup B, Yokoyama H, Kristiansen OP, Ostergaard PB, Olivecrona T (2004). Release of endothelium-associated proteins into blood by injection of heparin in normal subjects and in patients with Type 1 diabetes. Diabet. Med..

[23] Tloti MA, Moon DG, Weston LK, Kaplan JE (1991). Effect of 13-hydroxyoctadeca-9,11-dienoic acid (13-HODE) on thrombin induced platelet adherence to endothelial cells in vitro. Thromb. Res..

[24] Truitt A, McNeill G, Vanderhoek JY (1999). Antiplatelet effects of conjugated linoleic acid isomers. Biochim. Biophys. Acta.

[25] Greinacher A, Selleng K, Warkentin TE (2017). Autoimmune heparin-induced thrombocytopenia. J. Thromb. Haemost..

[26] Goodfriend TL (2007). Heparin, lipoproteins, and oxygenated fatty acids in blood: A cautionary note. Prostaglandins Leukot. Essent. Fatty Acids.

[27] Persson E (1988). Lipoprotein lipase, hepatic lipase and plasma lipolytic activity. Effects of heparin and a low molecular weight heparin fragment (Fragmin). Acta Med. Scand. Suppl..

[28] Tornvall P, Olivecrona G, Karpe F, Hamsten A, Olivecrona T (1995). Lipoprotein lipase mass and activity in plasma and their increase after heparin are separate parameters with different relations to plasma lipoproteins. Arterioscler. Thromb. Vasc. Biol..

[29] Gao C (2011). Heparin promotes platelet responsiveness by potentiating alphaIIbbeta3-mediated outside-in signaling. Blood.

[30] Yagi M (2012). Heparin modulates the conformation and signaling of platelet integrin alphaIIbbeta3. Thromb. Res..

[31] Wang B (2021). Metabolism pathways of arachidonic acids: Mechanisms and potential therapeutic targets. Signal Transduct. Target Ther..

[32] Payne DA, Jones CI, Hayes PD, Naylor AR, Goodall AH (2007). Therapeutic benefit of low-dose clopidogrel in patients undergoing carotid surgery is linked to variability in the platelet adenosine diphosphate response and patients' weight. Stroke.

[33] Janes SL, Wilson DJ, Chronos N, Goodall AH (1993). Evaluation of whole blood flow cytometric detection of platelet bound fibrinogen on normal subjects and patients with activated platelets. Thromb. Haemost..

[34] Thomas CP (2010). Phospholipid-esterified eicosanoids are generated in agonist-activated human platelets and enhance tissue factor-dependent thrombin generation. J. Biol. Chem..

