# Supplementary material for: Unfractionated heparin reverses aspirin inhibition of platelets during coronary artery bypass graft surgery
Source: Sci Rep. 2024 Apr 13;14:8572. doi: 10.1038/s41598-024-58005-x (PMC11015001; doi:10.1038/s41598-024-58005-x)
Supplement: Supplementary file 1 — Supplementary Information. [file 41598_2024_58005_MOESM1_ESM.pdf]

Unfractionated heparin reverses aspirin inhibition of platelets during coronary artery bypass graft surgery

Robert E Turnbull[1*], Azhar Hafeez[1], Katrin N Sander[2], David A Barrett[2], Gavin J Murphy[1], Alison H Goodall[1]

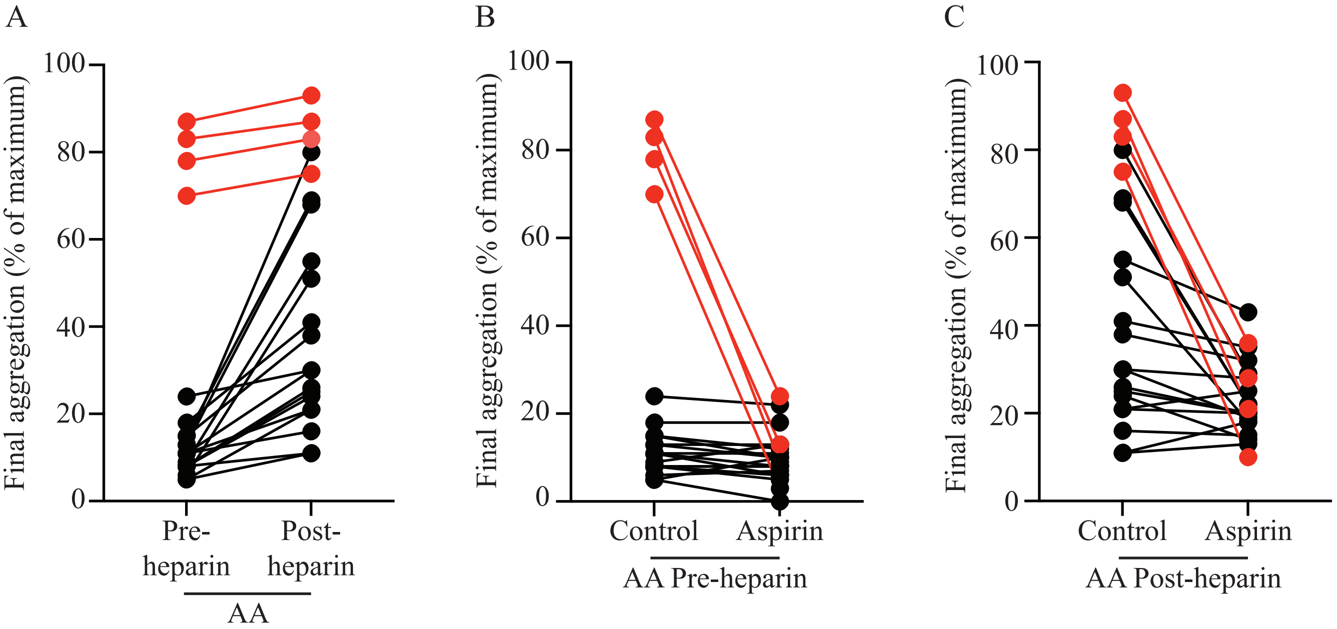

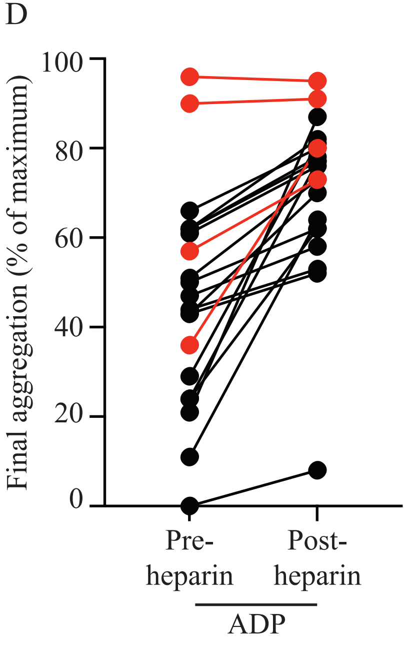

Supplementary Figure 1. Aggregation pre- and post-heparin infusion including the 4 patients excluded from the study due to apparent aspirin non-response. All four patients (shown in red) had >70% aggregation to AA pre-heparin compared to the 17 patients (in black) who were entered into the study (A). AA aggregation in these 4 patients was not significantly different post heparin (A and C) but was inhibited significantly by addition of aspirin in vitro to the pre-heparin sample (B). These data are consistent with aspirin non-compliance, or insensitivity. All 4 patients also had moderate to strong responses to ADP that were increased significantly after heparin infusion (D).

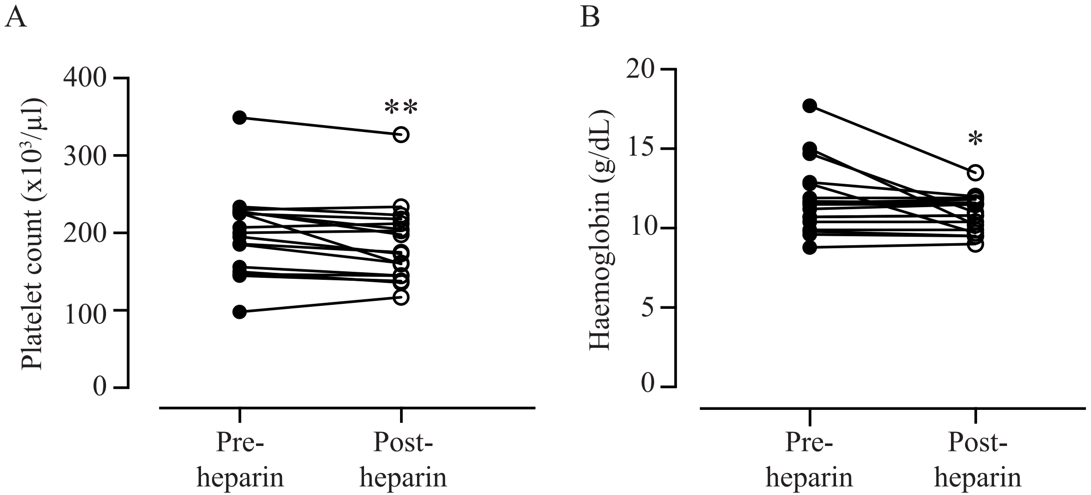

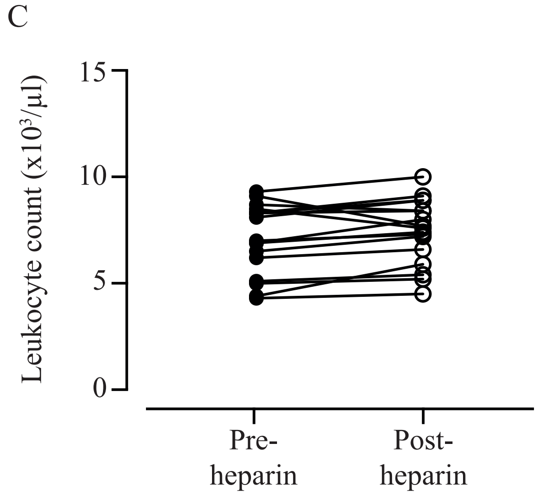

Supplementary Figure 2. Measurement of platelet count (a), heamoglobin (b) and leukocyte count (c) in whole blood from patients pre- and post-heparin. P-values calculated using Students' paired t-test (*<0.05, **<0.01), n=17.

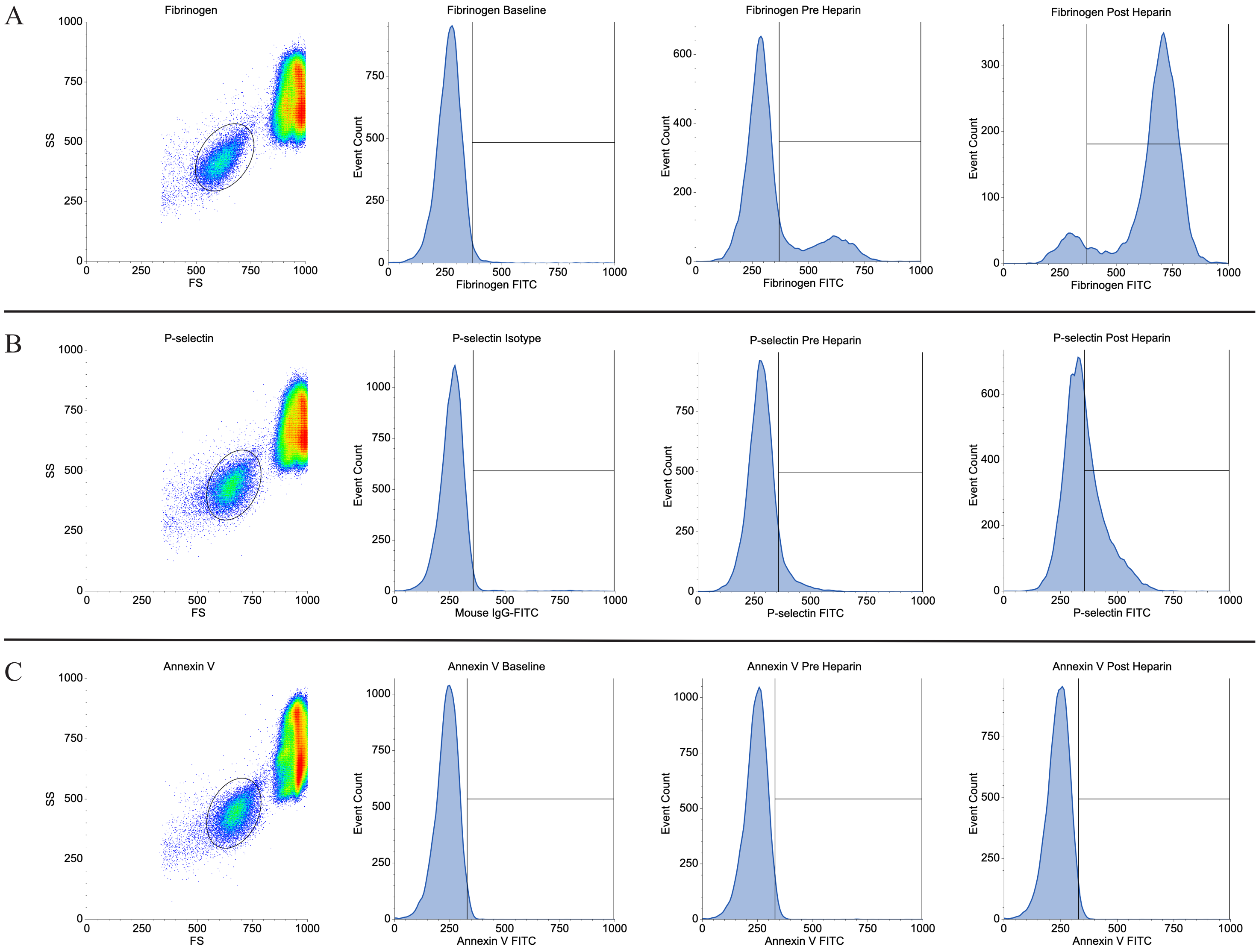

Supplementary Figure 3. Flow cytometric analysis of platelet activation in blood samples from patients. Examples of flow cytometric images to detect platelet (A) fibrinogen binding (B), P-selectin expression and (C) Annexin-V binding in whole blood from patients pre and post heparin. Examples of forward and side scatter (FS and SS) traces are shown in the left hand column. Platelets were gated by FS and SS and imaged for fluorescence intensity. Negative controls for fluorescence intensity (2nd column) were set at 2% against the relevant negative controls (see methods for details). Columns 3 and 4 show images obtained from patients' samples pre and post heparin infusion.

Supplementary Table 1. Correlations between patient pre- and post-aggregation, 12-HETE plasma levels and BMI

Parameter	AA aggn. post	ADP aggn. Post	Plasma [12-HETE] post	BMI
**Spontaneous aggn. post**				$R^2 = 0.2359$ $p = 0.0664$
**AA aggn. pre**	$R^2 = 0.2966$ $p = 0.0130$			$R^2 = 0.3220$ $p = 0.0274$
**AA aggn. post**	$R^2 = 1$	$R^2 = 0.2482$ $p = 0.0300$	$R^2 = 0.0419$ $p = 0.4470$	$R^2 = 0.3584$ $p = 0.0184$
**ADP aggn. pre**		$R^2 = 0.2135$ $p = 0.0403$		$R^2 = 0.4149$ $p = 0.0096$
**ADP aggn. post**		$R^2 = 1$	$R^2 = 0.0399$ $p = 0.4583$	$R^2 = 0.2847$ $p = 0.0405$
**Plasma [12-HETE] pre**			$R^2 = 0.2723$ $p = 0.0382$	
**Plasma [12-HETE] post**			$R^2 = 1$	
